# Desensitization of the Mechanoreceptors in Müller's Muscle Reduces the Increased Reflex Contraction of the Orbicularis Oculi Slow-Twitch Fibers in Blepharospasm

**Published:** 2014-09-12

**Authors:** Kiyoshi Matsuo, Ryokuya Ban, Midori Ban

**Affiliations:** Department of Plastic and Reconstructive Surgery, Shinshu University School of Medicine, Matsumoto, Japan

**Keywords:** orbicularis oculi reflex, trigemino-facial reflex, slow-twitch fibers, mechanoreceptors in Müller's muscle, blepharospasm

## Abstract

**Objective:** Although the mixed orbicularis oculi muscle lacks the muscle spindles required to induce reflex contraction of its slow-twitch fibers, the mechanoreceptors in Müller's muscle function as extrinsic mechanoreceptors to induce reflex contraction. We hypothesize that strong stretching of these mechanoreceptors increases reflex contraction of the orbicularis oculi slow-twitch muscle fibers, resulting in blepharospasm. **Methods:** We examined a 71-year-old man with right blepharospasm and bilateral aponeurosis-disinserted blepharoptosis to determine whether the patient's blepharospasm was worsened by increased trigeminal proprioceptive evocation via stretching of the mechanoreceptors in Müller's muscle owing to a 60° upward gaze and serrated eyelid closure, and whether local anesthesia of the mechanoreceptors via lidocaine administration to the upper fornix as well as surgical disinsertion of Müller's muscle from the tarsus and fixation of the disinserted aponeurosis to the tarsus decreased trigeminal proprioceptive evocation and improved patient's blepharospasm. **Results:** Before pharmacological desensitization, 60° upward gaze and serrated eyelid closure exacerbated the patient's blepharospasm. In contrast, these maneuvers did not worsen his blepharospasm following lidocaine administration. One year after surgical desensitization, the blepharospasm had disappeared and a 60° upward gaze did not induce blepharospasm. **Conclusions:** Strong stretching of the mechanoreceptors in Müller's muscle appeared to increase reflex contraction of the orbicularis oculi slow-twitch muscle fibers, resulting in blepharospasm. In addition to botulinum neurotoxin injections into the involuntarily contracted orbicularis oculi muscle and myectomy, surgical desensitization of the mechanoreceptors in Müller's muscle may represent an additional procedure to reduce blepharospasm.

Although the levator, frontalis, and orbicularis oculi muscles consist of fast-twitch and slow-twitch fibers (Fig 1a), these muscles lack the intrinsic muscle spindles required to induce reflex contraction of their slow-twitch fibers owing to proprioception evoked by stretching of mechanoreceptors in the muscle spindles.^1^^-^6 We previously reported that the mechanoreceptors in Müller's muscle functioned as extrinsic mechanoreceptors that induce reflex contraction of the levator, frontalis, and orbicularis oculi slow-twitch fibers via the trigeminal proprioceptive neurons in the mesencephalon.^7^^-^17

Furthermore, we previously reported that electrical stimulation of the trigeminal proprioceptive nerve innervating the mechanoreceptors in Müller's muscle induces reflex contraction of the orbicularis oculi slow-twitch muscle fibers in addition to the levator and frontalis slow-twitch muscle fibers (Fig 1a)^11^^,^^15^^,^^17^ and that a hydraulic mechanism caused by trauma to the globe impairs trigeminal proprioceptive evocation, which reduces reflex contraction of the levator and frontalis slow-twitch muscle fibers, resulting in eyelid and brow ptosis.^16^ We also revealed that trigeminal proprioception evoked by strong stretching of the mechanoreceptors in Müller's muscle due to upgaze with lid load induced reflex contraction of the orbicularis oculi slow-twitch fibers and that anesthesia of the mechanoreceptors in Müller's muscle precluded this reflex.^17^

Under these circumstances, we hypothesize that increased reflex contraction of the orbicularis oculi slow-twitch muscle fibers elicited by strong stretching of the mechanoreceptors in Müller's muscle may be a cause of blepharospasm and thus represent a therapeutic target. In this investigation, we clinically evaluated the possibility that reduced trigeminal proprioceptive achieved by desensitizing the mechanorceptors in Müller's muscle decreases reflex contraction of the orbicularis oculi slow-twitch muscle fibers in a patient with unilateral blepharospasm.

## METHODS

**Figure 1:**
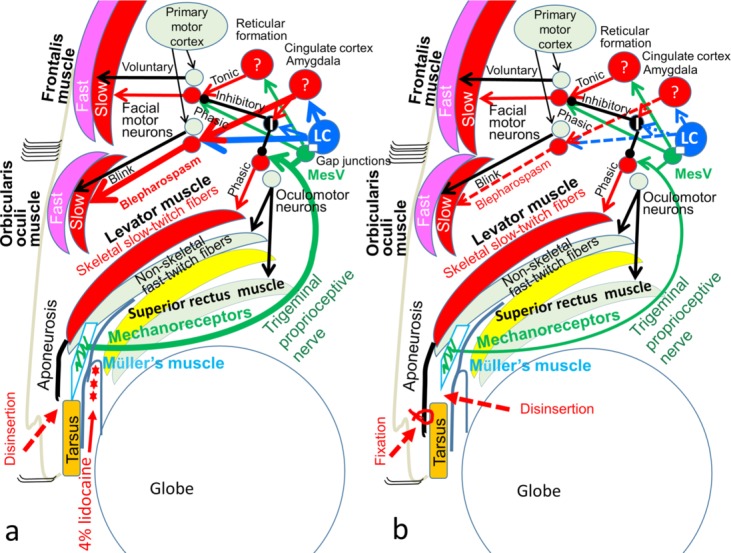
Neuroanatomy for the contraction of the levator, frontalis, and orbicularis oculi fast-twitch and slow-twitch muscle fibers. (*a*) Before surgery. Black arrows indicate voluntary contraction of the levator, frontalis, and orbicularis oculi fast-twitch muscle fibers. Red arrows indicate involuntary reflex contraction of the levator, frontalis, and orbicularis oculi fast-twitch muscle fibers. Green arrows indicate the proprioceptive nerve. Disinsertion indicates where the levator aponeurosis is disinserted from the tarsus. (*b*) After surgery. Disinsertion indicates where the Müller's muscle is disinserted from the tarsus; Fixation, where the disinserted aponeurosis is fixed to the tarsus; Question marks, an unknown nucleus at the reticular formation, cingulate cortex, or amygdala; Fast, fast-twitch muscle fibers; Slow, slow-twitch muscle fibers; mesV, mesencephalic trigeminal nucleus; LC, locus ceruleus; Phasic, phasic contraction; Reflex, reflex contraction; Tonic, tonic contraction; I, inhibitor neuron.

**Figure 4:**
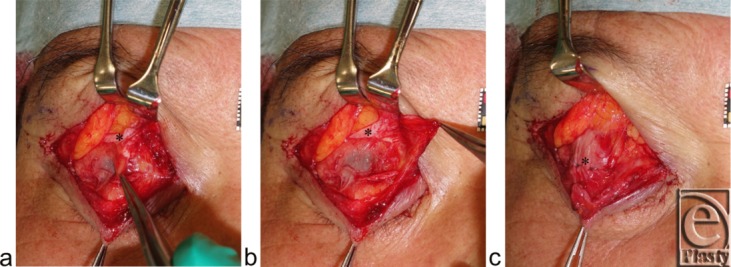
Surgical procedures (refer to Fig 1b). (*a*) Disinserting Müller's muscle from the tarsus. (*b*) The globe is visible through the conjunctiva palpebrae. (*c*) The disinserted aponeurosis is fixed to the tarsus with 3 stitches. Asterisks indicate the levator aponeurosis.

The patient was a 71-year-old man who sustained right blepharospasm and bilateral aponeurosis-disinserted blepharoptosis (Fig 1a). No blood vessel or tumor touching a facial nerve was detected via magnetic resonance imaging. First, to examine whether blepharospasm was worsened by increased trigeminal proprioceptive evocation via stretching of the mechanoreceptors in Müller's muscle, the patient was instructed to maintain a 60° upward gaze, lateral gazes, and serrated eyelid closure for several seconds (Fig 2). Second, to establish whether pharmacological reduction of trigeminal proprioceptive evocation via stretching of the mechanoreceptors in Müller's muscle improved blepharospasm, we administered 4% lidocaine to anesthetize the mechanoreceptors in the right Müller's muscle (Fig 1a). The patient was instructed to lie in a supine position, raise his chin, and gaze downward. Before administration of 4% lidocaine, 2 to 3 drops of 0.4% oxybuprocaine hydrochloride were placed on the surface of cornea and into the upper fornix. Next, the upper eyelid on the affected side was detached from the globe with a small retractor for 60 seconds to create a space in the upper fornix. Four to 5 drops of lidocaine were administered into the space and were retained in this position by gravity to exclusively anesthetize the mechanoreceptors in Müller's muscle. Soon afterwards, the patient was again asked to maintain a 60° upward gaze, lateral gazes, and serrated eyelid closure for several seconds (Fig 3). Finally, to demonstrate that surgical reduction of trigeminal proprioceptive evocation by stretching of the mechanoreceptors in Müller's muscle improves blepharospasm, the distal portion of Müller's muscle was disinserted from the tarsus on the right side and the disinserted aponeuroses were fixed to the tarsus on the right and left sides (Figs 1b and 4).^7^ One year postoperatively, the patient was again instructed to maintain a 60° upward gaze or lateral gazes for several seconds (Fig 5).

## RESULTS

Before pharmacological desensitization of the mechanoreceptors in Müller's muscle, 60° upward gaze, right lateral gaze, and serrated eyelid closure exacerbated the patient's blepharospasm (Fig 2). After pharmacological desensitization with lidocaine, the previous maneuvers did not worsen his blepharospasm (Fig 3). One year after surgical desensitization of the mechanoreceptors in Müller's muscle, the blepharospasm had disappeared, and 60° upward and right lateral gazes did not induce the blepharospasm (Fig 5).

## DISCUSSION

In our previous reports regarding reflex contraction of the levator, frontalis, and orbicularis oculi slow-twitch muscle fibers,^7^^-^17 we proposed that weak proprioceptive evocation via mild stretching of the mechanoreceptors in Müller's muscle in primary gaze would induce reflex contraction of the levator slow-twitch muscle fibers. We further proposed that moderate proprioceptive evocation by moderate stretching of these mechanoreceptors in upgaze would enhance reflex contraction of the levator slow-twitch muscle fibers and evoke reflex contraction of the frontalis slow-twitch muscle fibers, and that strong proprioceptive evocation through strong mechanoreceptor stretching, such as while yawning^18^ with increased contraction of the extraocular muscles including the levator nonskeletal fast-twitch muscle fibers to retract the globe backward, would induce reflex contraction of the orbicularis oculi slow-twitch muscle fibers (Fig 1a). Here, desensitization of the mechanoreceptors in Müller's muscle via local anesthesia or surgery reduced the increased reflex contraction of the orbicularis oculi slow-twitch fibers in blepharospasm and restored reflex contraction of the levator and frontalis slow-twitch fibers (Fig 1b). Our proposal therefore appears to be accurate. In addition, an interneuron may inhibit antagonizing neuromuscular units for reflex contraction of the levator and frontalis slow-twitch muscle fibers during increased reflex contraction of the orbicularis oculi slow-twitch muscle fibers (Fig 1a).^19^

Cortical control of eyelid closure is not well understood.^20^ Only recently has it been uncovered in primates that the cingulate cortex, and to lesser degree the primary cortex, are involved in this process.^21^ These findings were later confirmed in humans through transcranial magnetic stimulation mapping^22^ and functional magnetic resonance imaging.^23^ New findings also indicate major input from the amygdala, which presumably plays a role in behaviors such as emotional facial expressions.^24^ The locus ceruleus, which possibly connects with the mesencephalic trigeminal nucleus through gap junctions (Fig 1a),^13^ projects ascending axons to the forebrain, cingulate cortex, amygdala, and spinal motoneurons to facilitate muscle tone via involuntary contraction of skeletal slow-twitch muscle fibers.^25^^-^28 The locus ceruleus has been also reported to densely project to the facial motor neurons;^29^^,^^30^ this projection appears to be excitatory, because extracellular microiontophoretic application of norepinephrine increases the activity of facial motoneurons.^31^^-^34 Considering these results in combination with our results, we conclude that blepharospasm with increased reflex contraction of the orbicularis oculi slow-twitch muscle fibers may be caused by strong trigeminal proprioception stemming from strong stretching of the mechanoreceptors in Müller's muscle via excitation of the mesencephalic trigeminal nucleus, locus ceruleus, cingulate cortex, and/or amygdala (Fig 1).

Both upward gaze and right lateral gaze increased reflex contraction of the orbicularis oculi slow-twitch fibers in our patient. The levator and superior rectus muscles form an angle of 23° with the visual axis in primary gaze. Because the length of the levator muscle on the side of the lateral gaze is longer than that on the side of the medial gaze, the mechanoreceptors in Müller's muscle on the lateral gaze side appears to be have been stretched more. Accordingly, the stronger trigeminal proprioceptive evocation on the lateral gaze side may have enhanced reflex contraction of the orbicularis oculi slow-twitch muscle fibers more visibly than the weaker trigeminal proprioceptive evocation on the medial gaze side. Voluntary serrated eyelid closure appears to consist of increased voluntary contraction of the extraocular muscles, including the levator nonskeletal fast-twitch muscle fibers, strong trigeminal proprioception evocation by strong stretching of the mechanoreceptors in Müller's muscle, and subsequently increased involuntary reflex contraction of the orbicularis oculi slow-twitch muscle fibers. Predictably, voluntary serrated eyelid closure caused involuntary serrated eyelid closure as blepharospasm (Fig 2). As far as the unilaterality of the patient's blepharospasm is concerned, unilaterally increased neurocircuits among the extraocular muscles including the levator muscle, the mechanoreceptors in Müller's muscle, and the orbicularis oculi slow-twitch muscle fibers might exist in this patient.

In conclusion, in addition to botulinum neurotoxin injections into the involuntarily contracted orbicularis oculi muscle and myectomy,^20^ surgical desensitization of the mechanoreceptors in Müller's muscle may represent another procedure to reduce blepharospasm. This alternative approach may be especially useful in patients whose upward and lateral gazes and serrated eyelid closure accelerate involuntary reflex contraction of the orbicularis slow-twitch muscle fibers and in whom local anesthesia of the mechanoreceptors in Müller's muscle precludes contractions.

## Figures and Tables

**Figure 2:** A 71-year-old man exhibiting worsening blepharospasm after upgaze, right lateral gaze, or serrated eyelid closure.

**Figure 3:** After administration of 4% lidocaine into the right upper fornix, the patient's spasm was not worsened by upgaze, right lateral gaze, or serrated eyelid closure.

**Figure 5:** One year after surgery, blepharospasm is not induced by upgaze or right lateral gaze.
